# A new species of *Longitarsus* Latreille, 1829 (Coleoptera, Chrysomelidae, Galerucinae) pupating inside stem aerenchyma of the hydrophyte host from the Oriental Region

**DOI:** 10.3897/zookeys.87.1294

**Published:** 2011-03-24

**Authors:** K. D. Prathapan, C. A. Viraktamath

**Affiliations:** 1 Department of Entomology, Kerala Agricultural University, Vellayani P.O., Trivandrum - 695 522, Kerala, India; 2 Department of Entomology, University of Agricultural Sciences, GKVK P.O., Bangalore - 560 065, India

**Keywords:** Chrysomelidae, subaquatic, *Longitarsus*, new species, key, stem aerenchyma pupation, *Limnophila*

## Abstract

A new species of subaquatic *Longitarsus* pupating inside the stem aerenchyma of its hydrophyte host plant is described. Eggs are laid on tender leaves and buds and the larvae are open feeders. This is the first report of an Oriental flea beetle pupating inside the stem of its hydrophyte host. A key to the species of southern Indian *Longitarsus* is provided.

## Introduction

Larvae of flea beetles, in general, are subterranean root feeders. A few of them mine the leaves or feed exposed on it, while fruit borers and stem borers are extremely rare (Jolivet and Hawkeswood 1995, Konstantinov and Vandenberg 1996). As a rule, flea beetle larvae associated with terrestrial plants, including leaf feeders, pupate in soil or litter. However, root and soil under submergence are inaccessible to all life stages of flea beetles due to lack of adaptation for a true aquatic life. Still a few of them have adopted strategies to circumvent this limitation to harness aquatic and subaquatic plants. Jolivet (2003) reviewed the biology of subaquatic Chrysomelidae, including subaquatic flea beetles. In many such flea beetles, larvae feed on leaves and pupation occurs in the soil on shore or facultatively on the plant itself when soil is not available. In the case of beetles living on rooted emergents and floating plants, inaccessibility to soil force all life stages, including pupae, to adapt to the portions of the plant above water level. For example, *Pseudolampsis guttata* (LeConte) that feeds on the floating water fern *Azolla caroliniana* Willd. pupates on leaf surface (Buckingham and Buckingham 1981). Species of *Agasicles* Jacoby, 1904 are unique among the subaquatic flea beetles as they pupate only inside the stem internode cavity of their amaranthaceous hosts (Vogt et al. 1979). A pair of modified T-shaped urogomphi secures the pupa from falling into the wedged position inside the internode cavity, from which emergence might be impossible (Vogt et al. 1979; Cox 1998). A new species of *Longitarsus* Latreille, 1829 from the Oriental Region, similarly adapted for pupation in stem aerenchyma is described here. Aerenchyma is a large intercellular space that acts as a mediator of internal gas exchange and maintains strength with the least tissue (Jung et al. 2008).

The cosmopolitan *Longitarsus* isthe most speciose genus of flea beetles with about 700 species. Though about 100 species are known from the Oriental Region, only eight named species of *Longitarsus* occur in south India (Maulik 1926; Scherer 1969; Gruev and Askevold 1988; Prathapan et al. 2005) and several of them still await naming and description. Known host plants of the genus belong to at least a dozen families with marked preference for members of Boraginaceae, Lamiaceae, Asteraceae, and Verbenaceae (Jolivet and Hawkeswood 1995). Species of *Longitarsus* are small to medium sized flea beetles easily identified by a very long first metatarsomere which is at least half as long as the metatibia. Other salient features of the genus are the lack of transverse or longitudinal impressions on the pronotum, open procoxal cavities and the confused elytral punctures rarely forming regular striae. Members of *Longitarsus* are generally terrestrial and their larvae feed on the roots (Furth 1980, Ireson et al. 1991, Schwarzländer 2000, Baars 2001 and Simelane 2010). Exceptions include the European *Longitarsus nigerrimus* (Gryllenhal) that lives in peat bogs and swamps. Booth (2000) reviewed its records from the British Isles and Jolivet (2003) summarized information on its biology.

## Methods

Natural populations of the beetle under field conditions were observed during 2009–2011 at Vembayam, Trivandrum, Kerala, India during several visits. The host plant, *Limnophila aquatica* (Roxb.) Alston was grown in a concrete tank partially filled with soil and water at Vellayani and live beetles were released on to these plants to confirm the biology observed in the field.

Descriptive terminology follows Konstantinov (1998). The holotype of the new species is deposited in the Natural History Museum, London (BMNH). Paratypes will be deposited in the National Pusa Collection, Indian Agricultural Research Institute, New Delhi (NPC), University of Agricultural Sciences, Bangalore (UASB), National Museum of Natural History, Smithsonian Institution, Washington DC (USNM), and in the personal collection of the first author (PKDC). Plant vouchers are deposited in the Calicut University Herbarium (Accession nos 6426, 6427, 113059, 113060). The immature stages are being retained by the first author for further studies.

## Systematics

### Longitarsus limnophilae

Prathapan & Viraktamath sp. n.

urn:lsid:zoobank.org:act:A24AE7BC-6B6D-4880-994E-BB8D05667079

Figs 1 8 12 15 

#### Holotype

♂, with labels as follows: “INDIA Kerala / Vembayam / 12. ix. 2009 Prathapan Coll.” “*Longitarsus limnophilae* sp. nov. / Prathapan & Viraktamath” “HOLOTYPE [red printed label]” (BMNH).

#### Paratypes

(30 specimens): 7 ♂, 3 ♀. The same labels as holotype; 5 ♀. same data as for holotype except dating 3.x.2009; 5 ♂. same data except dating 24.x.2009; 9 ♂, 1 ♀. same data except dating 16.i.2010 (5 BMNH, 5 USNM, 5 UASB, 12 NPC, 3 PKDC).

**Figure 1.**
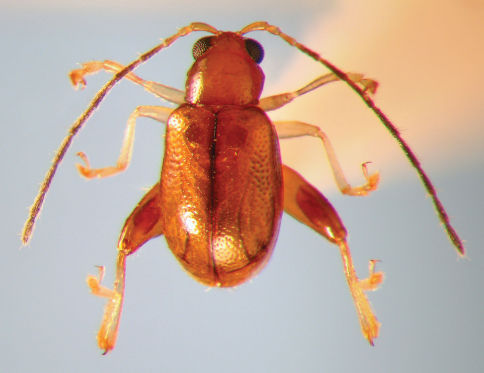
*Longitarsus limnophilae* sp. n., dorsal habitus

#### Etymology.

This unique species is named after its host plant. The name is a noun in the genitive case.

#### Description.

Length 1.89 – 2.15 mm; width 0.91 – 1.08 mm; female (2.09 – 2.15 mm) slightly larger than male (1.89 – 2.12 mm). General color brown (Fig. 1). Fore- and middle legs, hind tibia and tarsi light brown. Antenna piceous with proximal three to five antennomeres gradually turning brown. Labrum dark brown to piceous, suture narrowly piceous. Ventrites lighter than dorsum.

**Figures 2–8.**
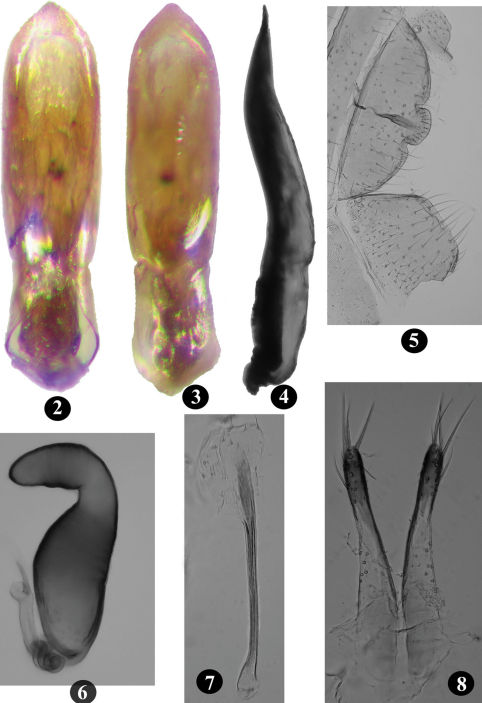
*Longitarsus limnophilae* sp. n. **2** median lobe of aedeagus, ventral view; **3** median lobe of aedeagus, dorsal view; **4** median lobe of aedeagus, lateral view; **5** last abdominal ventrite of male (macerated specimen); **6** spermatheca; **7** tignum; **8** vaginal palpi.

Vertex shiny, impunctate, minutely wrinkled. Ommatidia fully developed. Postcallinal sulcus weak but distinct. Frontal ridge unusual, broad and not sharply raised, anteriorly widening towards frontoclypeal suture, anteriorly forming ill-defined denticle in middle of flat, poorly developed anterofrontal ridge. Maxillary palpus with last palpomere longest. Antenna extends well beyond apex of elytra over pronotum. Second antennomere longer than half of third; second and third together longer than first, subequl to fourth; fifth longer than fourth; fifth to seventh subequal, eighth to tenth progressively shorter than previous antennomere. Pronotum anteriorly wider than posteriorly; 1.27 – 1.34 times wider than long; anterolateral callosity posteriorly lower than anteriorly, not forming denticle at pore; posterolateral callosity protrudes beyond lateral margin; lateral margin weakly curved, anteriorly broader than posteriorly; disc shiny with minute punctures more evident posteriorly. Elytra with well developed humeral calli, punctures distinct, width of interstices smaller than diameter of one puncture in middle of elytron. Elytral apex convex, with one long seta. Hind wings well developed. Scutellum triangular. First male protarsomere 1.60 – 1.67 times longer than wide; first female protarsomere 2.00 – 2.43 times longer than wide. Metatibia strongly curved in dorsal view, slightly curved in lateral view. Number of spinules on dorsolateral margin of metatibia, proximal to row of stiff bristles, vary from seven to ten. In lateral view, first metatarsomere 0.55 – 0.57 times as long as metatibia. Proximal end of first metatarsomere ventrally with thick characteristic patch of short pointed and capitate setae in both sexes. Last male ventrite internally with longitudinal ridge along middle (Fig. 5); posterior margin bisinuate.

Aedeagus in lateral view gently curved, apex acutely pointed and slightly recurved (Fig. 4); ventral side depressed with transparent window, lateral edges raised (Fig. 2); dorsal opening covered with lamina not extending to apex (Fig. 3). Arms of tegmen subequal to or slightly shorter than stem.

Spermatheca with receptacle widest in middle, internal side strongly convex, external side weakly concave; pump with horizontal part longer than vertical; spermathecal duct curved towards receptacle, coiled thrice proximally, not reaching half of receptacle (Fig. 6). Vaginal palpus narrow at distal 1/3, widest at proximal 1/4; distal sclerotization shorter than proximal sclerotization or lateral membranous area (Fig. 8). Tignum nearly straight, anterior sclerotization slightly wider than posterior (Fig. 7).

**Figures 9–12.**
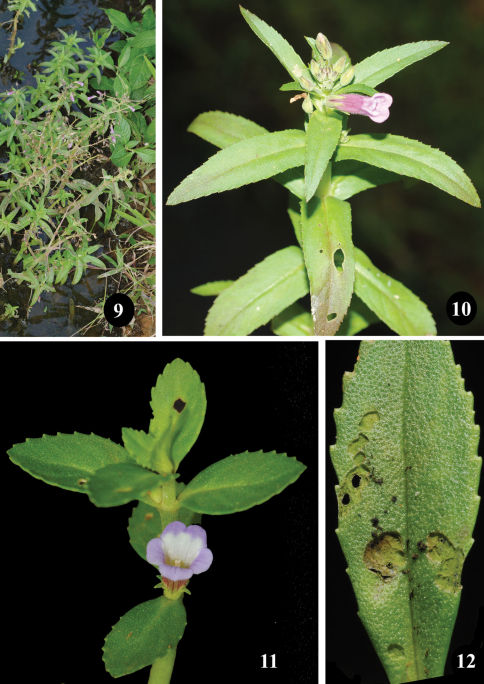
*Longitarsus limnophilae* sp. n. **9** habitat; **10** *Limnophila aquatica*; **11** *Longitarsus repens*; **12** adult feeding scars on leaf.

**Figures 13–16.**
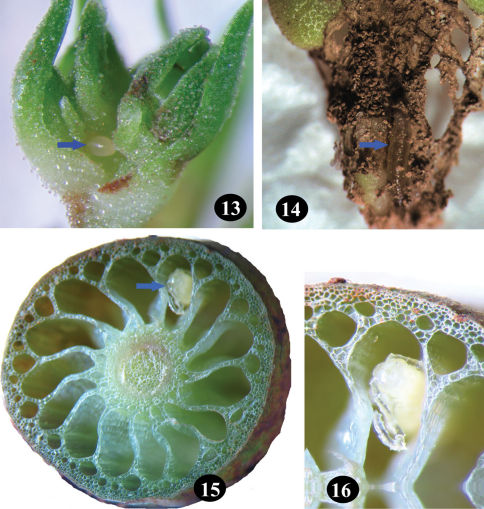
*Longitarsus limnophilae* sp. n. **13** egg; **14** larva; **15, 16** pupa inside stem aerenchyma.

#### Remarks.

*Longitarsus limnophilae* can easily be separated from all other south Indian species of *Longitarsus* by the anteriorly widening, flat frontal ridge (in the other species, the frontal ridge is more or less narrowly raised). *Longitarsus belgaumensis* Jacoby closely resembles *Longitarsus limnophilae* in having narrowly piceous elytral suture and dark distal antennomeres. But *Longitarsus limnophilae* can be separated from *Longitarsus belgaumensis* based on the antenna extending slightly beyond the apex of elytra over pronotum (in *Longitarsus belgaumensis*, antenna does not extend beyond the apex of elytra over pronotum), pronotum anteriorly wider than posteriorly (in *Longitarsus belgaumensis*, pronotum is anteriorly as wide as posteriorly with the maximum width in the middle), structure of frontal ridge (frontal ridge sharply raised along middle in *Longitarsus belgaumensis*) and genitalia.

#### Host plants.

*Limnophila aquatica* (Roxb.) Alston (Scrophulariaceae) (Fig. 10) is a rooted emergent hydrophyte growing in shallow streams, marshes and rice fields (Fig. 9). Species of *Limnophila* R. Br., 1810 are widely distributed in the tropics and subtropics of the Old World and also occur as weeds. Stem aerenchyma in *Longitarsus aquatica* resembles the same in *Longitarsus sessiliflora* (Vahl) Blume termed as “wheel-type” by Jung et al. (2008). *Longitarsus limnophilae* was also found to feed on *Longitarsus repens* (Benth.) Benth. (Fig. 11).

#### Biology.

Eggs (Fig. 13) are laid on tender leaves and buds and the larvae are open feeders (Fig. 14). The closely oriented tender leaves provide sufficient cover for the larva. Mature larva enters the stem aerenchyma in the internode by boring a tiny hole and pupation occurs in it, a little above the entry hole (Figs 15, 16). Adult emerges through an exit hole nearly circular in shape with irregular margin. Adult feeds on both adaxial and abaxial surface of, mostly, tender leaves by scraping, often resulting in holes on the lamina (Fig. 12). Adults when thrown in water floated initially and then swam with raised antennae held back over the sides of the pronotum. After swimming for a while, some performed a short jump on to the shore.

#### Distribution.

The types were collected from a single locality only. India (Kerala, Vembayam, 8°38'28"N, 76°56'39"E).

#### Discussion.

Jolivet and Hawkeswood (1995) have listed *Bacopa* Aublet, 1775 (Scrophulariaceae), a genus of hydrophytes, among host plants of *Longitarsus*. But no further information is available on its biology on *Bacopa*. *Longitarsus limnophilae* and species of *Agasicles* represent two independently evolved lineages in Alticini adapted for larval leaf feeding and pupation inside the stem. *Agasicles* and *Longitarsus limnophilae* are the only flea beetles known to pupate inside the stem of their aquatic host plant above the water level. Wheel shaped stem aerenchyma of *Limnophila aquatica* serve as a safe abode for the pupa offering protection against natural enemies. Species of *Limnophila* being widely distributed aquatic weeds, *Longitarsus limnophilae* could be a potential biocontrol agent for them.

This is the first report of an Oriental flea beetle pupating inside the stem of its hydrophyte host.

### Key to the southern Indian species of *Limnophila*

**Table:** 

1	Vertex with conspicuous deep punctures	*Longitarsus rangoonensis* Jacoby
–	Vertex impunctate to minutely punctuate	2
2	Elytra laterally with prominent longitudinal ridge extending backwards from the humerus	*Longitarsus liratus* Maulik
–	Elytra laterally without longitudinal ridge extending from humerus	3
3	Metatibial spur minutely serrulated on either side dorsally	*Longitarsus serrulatus* Prathapan, Faizal & Anith
–	Metatibial spur not serrulated	4
4	Elytral suture narrowly piceous compared to rest of elytra	5
–	Elytral suture not distinctly darker than rest of elytra	6
5	Frontal ridge broad, widening towards frontoclypeal suture, not sharply raised; antenna extends well beyond apex of elytra over pronotum	*Longitarsus limnophilae* Prathapan & Viraktamath,sp. n.
–	Frontal ridge sharply raised, narrow; antenna hardly reaches apex of elytra over pronotum	*Longitarsus belgaumensis* Jacoby
6	Dorsum red	*Longitarsus rufipennis* Jacoby
–	Dorsum yellow brown or dark brown	7
7	Three basal and four apical antennomeres light brown, four intermediate antennomeres dark brown to piceous	*Longitarsus gilli* Gruev & Askevold
–	Middle antennomeres not distinctly darker than distal or basal antennomeres	8
8	Dorsum uniform light brown; hind wings well developed; elytral punctures tend to be regular	*Longitarsus sari* Maulik
–	Elytra dark brown with lighter margins; hind wings absent; punctures confused	*Longitarsus fumidus* Maulik

## Supplementary Material

XML Treatment for Longitarsus limnophilae

## References

[B1] Baars JR (2001) Biology and laboratory culturing of the root-feeding flea beetle, *Longitarsus columbicus columbicus* Harold, 1876 (Chrysomelidae: Alticinae): A potential natural enemy of *Lantana camara* L. (Verbenaceae) in South Africa. Entomotropica 16:149-155

[B2] Booth RG (2000) A review of *Longitarsus nigerrimus* (Gryllenhal) (Chrysomelidae) records from the British Isles. The Coleopterist 9:15-18

[B3] Buckingham RG Buckingham M (1981) A laboratory biology of *Pseudolampsis guttata* (LeConte) (Coleoptera: Chrysomelidae) on waterfern, *Azolla caroliniana* Willd. (Pterydophyta: Azollaceae). The Coleopterists Bulletin 35:181-188

[B4] Cox ML (1998) The pupae of Chrysomeloidea and their use in Phylogeny (Coleoptera). In: Biondi M Daccordi M Furth DG (Eds) Fourth International Symposium on the Chrysomeldiae Proceedings of XX I. C. E. Firenze, 1996. Museo Regionale Di Scienze Naturali, Florence, 73–90

[B5] Furth DG (1980) Wing polymorphism, host plant ecology, and biogeography of *Longitarsus* in Israel (Coleoptera: Chrysomelidae). Journal of Entomology 13:125-148

[B6] Gruev B Askevold IS (1988)Pan-Pacific Entomologist 64:139-145

[B7] Ireson JE Friend DA Holloway RJ Paterson SC (1991) Biology of *Longitarsus flavicornis* (Stephens) (Coleoptera: Chrysomelidae) and its effectiveness in controlling ragwort (*Senecio jacobaea* L.) in Tasmania. Journal of Australian Entomological Society 30:129-141

[B8] Jolivet P (2003) Subaquatic Chrysomelidae. In: Furth DG (Ed) Special Topics in Leaf Beetle Biology. Proceedings of the 5th International Symposium on the Chrysomelidae. Pensoft Publishers, Sofia–Moscow, 303–332

[B9] Jolivet P Hawkeswood TJ (1995) Host-plants of Chrysomelidae of the world: An Essay about the Relationships between the Leaf-beetles and their Food-plants. Backhuys Publishers, Leiden, 281 pp.

[B10] Jung J Lee SC Choi HK (2008) Anatomical Patterns of Aerenchyma in Aquatic and Wetland Plants. Journal of Plant Biology 51:428-439

[B11] Konstantinov AS (1998) Revision of the Palearctic species of *Aphthona* Chevrolat and cladistic classification of the Aphthonini (Coleoptera: Chrysomelidae: Alticinae). Memoirs on Entomology, International. Associated Publishers, Gainesville, 429 pp.

[B12] Konstantinov AS Vandenberg NJ (1996) Handbook of Palearctic flea beetles (Coleoptera: Chrysomelidae: Alticinae). Contributions on Entomology, International 1:237-439

[B13] Maulik S (1926) The Fauna of British India including Burma and Ceylon. Coleoptera. Chrysomelidae. (Chrysomelinae and Halticinae). Taylor and Francis, London, 442 pp.

[B14] Prathapan KD Faizal MH Anith KN (2005) A new species of *Longitarsus* (Coleoptera: Chrysomelidae) feeding on Chinese potato, *Plectranthus rotundifolius* (Lamiaceae) in southern India. Zootaxa 966:1-8

[B15] Scherer G (1969) Die Alticinae des indischen Subkontinentes. Pacific Insects Monograph 22:1-251

[B16] Schwarzländer M (2000) Host specificity of *Longitarsus quadriguttatus* Pont., a below-ground herbivore for the biological control of Houndstongue. Biological Control 18:18-26

[B17] Simelane DO (2010) Potential impact of an introduced root-feeding flea beetle, *Longitarsus bethae*, on the growth and reproduction of an invasive weed, *Lantana camara*. Biological Control 54:114-118

[B18] Vogt GB McGuire Jr JU Cushman AD (1979) Probable Evolution and Morphological Variation in South American Disonychine Flea Beetles (Coleoptera: Chrysomelidae) and Their Amaranthaceous Hosts. U. S. Dep. Agric. Tech. Bull. No. 1593. USDA, Washington, DC, 148 pp.

